# Abeta is prerequisite, but insufficient to cause tau phosphorylation *in vivo*: tau phosphorylation in APP mice by diabetes

**DOI:** 10.1186/1750-1326-8-S1-P35

**Published:** 2013-09-13

**Authors:** Naoyuki Sato

**Affiliations:** 1 Department of Clinical Gene Therapy, Department of Geriatric Medicine, Suita, Osaka, Japan

## Background

Epidemiological studies suggest that diabetes mellitus increases the risk of onset of Alzheimer disease (AD). However, the underlying mechanisms have not been fully understood. Retrospective studies indicate that diabetes does not increase senile plaques. On the other hand, a Japanese cohort, Hisayama study suggested that insulin resistance in midlife increased the risk of development of senile plaques. Therefore, the aim of this study is to understand the mechanisms by which diabetes increase the risk of AD by dividing them into two phases; 1) before and 2) after the development of senile plaques.

## Methods

1) To test whether insulin resistance increases Abeta accumulation in the brain, we fed wild-type mice with a high-fat diet and measured the levels of Abeta in the brain. 2) To investigate the effects of diabetes on AD, we further analyzed the phenotypes of APP+*ob/ob* mice [1], which showed the increased cerebral amyloid angiopathy and impaired insulin signaling, especially focusing on tau phosphorylation.

## Results

1) Six months on a high fat diet increased Abeta40 in B6C mice brain. 2) 18 month old APP+*ob/ob* mice showed highly increased level of tau phosphorylation in the brain. Furthermore, a high fat diet increased plasma Abeta levels in APP/PS1 mice, but not in wild type mice.

## Conclusion

Tau phosphorylation is increased by diabetes in APP mice, suggesting that Abeta is prerequisite, but insufficient to cause tau phosphorylation *in vivo.* Abeta accumulation, insulin signaling and tau phosphorylation might play essential roles in the pathological interaction between AD and diabetes [2]. Of note, a vicious cycle likely underlies the interaction between AD and diabetes. High fat diet-induced elevation of plasma Abeta level might be involved in this mutual pathological interaction between the diseases [3,4]. These results suggest that diabetes disrupts homeostasis against AD.

## References

[B1] Shuko Takeda Naoyuki Sato Diabetes accelerated memory dysfunction via cerebrovascular inflammation and Abeta deposition in an Alzheimer mouse model with diabetes Proc Natl Acad Sci U S A 2010 107 7036 7041 10.1073/pnas.1000645107 20231468 PMC2872449

[B2] Naoyuki Sato Role of insulin signaling in the interaction between Alzheimer disease and diabetes mellitus: a missing link to therapeutic potential Current Aging Science 2011 4 118 127 10.2174/1874609811104020118 21235496

[B3] Shuko Takeda Naoyuki Sato Oral Glucose Loading Modulates Plasma Abeta Level in Alzheimer Disease Patients: Potential Diagnostic Method for Alzheimer Disease Dementia and Geriatric Cognitive Disorders 2012 34 25 30 10.1159/000338704 22889768

[B4] Naoyuki Sato Plasma Abeta: A Possible Missing Link Between Alzheimer Disease and Diabetes Diabetes 2013 62 1005 1006 10.2337/db12-1549 23520271 PMC3609572

